# Programmed expression of pro-apoptotic BMCC1 during apoptosis, triggered by DNA damage in neuroblastoma cells

**DOI:** 10.1186/s12885-019-5772-4

**Published:** 2019-06-06

**Authors:** Mohammad Sazzadul Islam, Ryo Takano, Tomoki Yokochi, Jesmin Akter, Yohko Nakamura, Akira Nakagawara, Yasutoshi Tatsumi

**Affiliations:** 1 Division of Innovative Cancer Therapeutics, Chiba Cancer Center Research Institute, Chiba, Japan; 2 Division of Oncogenomics, Chiba Cancer Center Research Institute, Chiba, Japan; 3 Division of Cancer Registry, Prevention and Epidemiology, Chiba Cancer Center Research Institute, Chiba, Japan; 4 Department of Molecular Biology and Oncology, Chiba University Graduate School of Medicine, Chiba, Japan; 5 Department of Clinical Research, Chiba Tokushukai Hospital, Chiba, Japan; 6 SAGA HIMAT Foundation, Saga, Japan

**Keywords:** BMCC1, E2F1, Apoptosis, Caspase-9, DNA damage, Neuroblastoma

## Abstract

**Background:**

The multi-functional BMCC1 (BCH motif-containing molecule at the carboxyl terminal region 1)/PRUNE2 plays a clear role in suppression of tumor activity. In the patients with neuroblastoma (NB), reduced expression of *BMCC1* in primary tumor tissues was associated with poor prognosis. By contrast, enforced expression of BMCC1 as well as elevated expression of BMCC1 in response to DNA-damage promotes apoptosis by abrogating Akt-mediated survival pathways.

**Methods:**

We addressed molecular mechanisms underlying changes in regulation of BMCC1 expression during the process of apoptosis, which was promoted by a DNA-damaging drug Cisplatin (CDDP), in NB-derived cells.

**Results:**

Elevated expression of BMCC1 was identified as an early response to DNA damage, which is accompanied by phosphorylation of ataxia telangiectasia mutated kinase (ATM) and accumulation of E2F1. Indeed, inhibition of ATM using an ATM inhibitor resulted in a decrease in expression of *BMCC1* at mRNA levels. In addition, an E2F-binding sight was required for activation of *BMCC1* promoter in response to DNA damage. On the other hand, knockdown of E2F1 yielded abrogated induction of BMCC1 in the cells after treatment with CDDP, suggesting that BMCC1 accumulation was caused by ATM-E2F1-dependent transcription. Finally, we demonstrated that full-length BMCC1 was proteolytically cleaved by apoptosis-activated caspase-9 during advanced stages of apoptosis in SK-N-AS cells.

**Conclusions:**

In this study, we demonstrated the programmed expression of full-length BMCC1 in human NB cells undergoing DNA damage-induced apoptosis. The elucidation of the molecular mechanisms controlling the regulation of BMCC1 during apoptosis initiated by DNA damage provides useful information for understanding drug resistance of tumor cells and spontaneous regression of NB.

**Electronic supplementary material:**

The online version of this article (10.1186/s12885-019-5772-4) contains supplementary material, which is available to authorized users.

## Background

Neuroblastoma (NB) is a common malignant solid tumor in children, which originates from the sympathoadrenal lineage of neural crest. When NB is diagnosed at age less than one year, it can regress spontaneously (even for the metastatic stage), resulting in a favorable prognosis [1]. However, details of the molecular mechanism responsible for this spontaneous regression remain obscure [2].

Human *BMCC1* (*B*CH *m*otif-*c*ontaining molecule at the *c*arboxyl terminal region *1*)/*PRUNE2* encodes a 340-kDa protein with a conserved BNIP-2 and Cdc42GAP homology (BCH) scaffold domain on its C-terminus [3–5]. Previous studies have demonstrated that BCH domain can modulate signaling networks and affect multiple cellular functions, such as morphogenesis, differentiation, motility, and apoptosis [4]. Therefore, functional contributions of BMCC1 in the regulation of signaling networks and multiple cellular functions, including apoptosis, have been suggested.

Our previous research has demonstrated that BMCC1 promotes neuronal apoptosis caused by nerve growth factor (NGF)-depletion [3]. In addition, BMCC1 can initiate and promote apoptosis via its C-terminal BNIP-2 homology region by inhibiting multiple steps in Akt-mediated survival pathway, as observed in NB and non-NB cells [5]. Increased expression of BMCC1 in response to DNA damage has linked to phosphorylation of ataxia telangiectasia mutated (ATM) at Ser-1981, corresponding to its sensitivity to DNA-damaging drugs [5]. Recently, we found that *BMCC1* was trans-activated by E2F1 during normal cell cycle by binding to *BMCC1* promoter in human cells [6]. However, precise expression profile of BMCC1 and effects on the apoptotic process initiated by DNA damage remain unclear.

E2F transcription factors can modulate expression of a series of genes associated with cell cycle progression, apoptosis, and DNA repair [7]. Among the E2F family proteins, molecular contribution of E2F1 in the progression of apoptosis caused by DNA damage has been characterized. In apoptotic cells triggered by DNA damage, E2F1 accumulates by stabilization of protein levels and not by transcriptional induction of mRNA levels [8–12]. Stabilization and subsequent activation of E2F1 following DNA damage can be partially modulated by DNA damage-phosphorylated ATM/ATM, RAD3-related (ATR), and Chk2 [12–14], which results in elevated expression of pro-apoptotic transcription targets, such as *p73* [15–17] and *Apaf1* [18].

DNA damage facilitates accumulation of intrinsic apoptosis [19–21], which promotes breakdown of mitochondrial membrane and cytosolic release of cytochrome *C*. Then it can bind to Apaf1 to induce activation of caspase-9 through proteolytic cleavage [22]. Subsequently, downstream caspases, such as caspase-3, caspase-6, and caspase-7, are activated through their proteolytic cleavage by active caspase-9 [23]. Similarly, the BNIP family proteins, which include BNIP-2 and BNIP-XL, are also cleaved by in vitro activated caspases, although their functional contribution to apoptotic process was not elucidated [24].

In this study, regulatory mechanism of BMCC1 in apoptosis of human cells initiated by Cisplatin (CDDP) was investigated. Elevated expression of full-length BMCC1 associated with early responses to DNA damage and reduced expression of BMCC1 in apoptotic cells were found. A further analysis demonstrated that acute induction of *BMCC1* at transcriptional levels was mediated by E2F1. Subsequent reduction of BMCC1 during apoptosis was due to the proteolytic cleavage by caspase-9. The findings of this study provide evidence for a better understanding of the tumor suppressive function of BMCC1, as a signal for apoptosis of cells with DNA damage.

## Materials and methods

### Cell culture, treatments, cell viability, knockdown and overexpression

Human NB-derived SK-N-AS, NBL-S, and NLF cells were obtained from the Children’s Hospital of Philadelphia cell line bank and were maintained in RPMI 1640 culture medium (Wako, Osaka, Japan), supplemented with 10% heat-inactivated fetal bovine serum (FBS, Thermo Fisher Scientific, Waltham, MA) and 100 U/mL Penicillin-Streptomycin (Thermo Fisher Scientific). LNCaP cells were provided from Dr. Ueda [5, 25] and were cultured in RPMI 1640 medium supplemented with 5% heat-inactivated FBS and 100 U/mL Penicillin-Streptomycin. The cells were grown in 5% CO_2_ and humid condition at 37 °C [5]. Cisplatin (CDDP, Sigma-Aldrich, St. Louis, MO) was used to induce DNA damage, and 2-Morpholin-a-yl-6-thianthren-1-yl-pyran-4-one (ATM kinase inhibitor, Calbiochem, San Diego, CA) was used to inhibit ATM activity. To inhibit the activity of caspases, SK-N-AS cells were treated with 40 μM of CDDP and were cultured in the presence of 50 μM of a pan-caspase inhibitor (Z-VAD-FMK, Promega, Madison, WI) or a caspase-9-specific inhibitor (Z-LEHD-FMK, R&D systems, Minneapolis, MN). After 48 h of CDDP treatment, cell viability was measured using WST-8 assay kit (Dojindo, Kumamoto, Japan), and yielded absorbance on 450 nM was detected with Microplate Reader MTP-310 (HITACHI, Tokyo, Japan). For knockdown of E2F1 using lentiviral shRNA delivery system, MISSION shRNA plasmid DNA clones (shE2F1#1 (TRC 0000000251) and shE2F1#2 (TRC 0000000252)) were purchased from Sigma-Aldrich.

### Semiquantitative RT-PCR and quantitative real-time PCR

Total RNA was extracted from NB cell lines using RNeasy Mini Kit (Qiagen, Hilden, Germany). The cDNA was reverse-transcribed from total RNA using SuperScript II reverse transcriptase and random primers (Thermo Fisher Scientific) and subjected to PCR-based amplification using rTaq DNA polymerase (TaKaRa, Shiga, Japan). The primer sets used for PCR were as follows:

*BMCC1* forward: 5′- GAAGCCTCTGGTCCAGTCAG-3′;

*BMCC1* reverse: 5′-CTTCGGCCGTATATTCTGGA-3′;

*E2F1* forward: 5′-TGCAGAGCAGATGGTTATGG-3′;

*E2F1* reverse: 5′-GTTCTTGCTCCAGGCTGAGT-3′;

*TAp73* forward: 5′-CCTCTGGAGCTCTCTGGAAC-3′;

*TAp73* reverse: 5′-GAAGACGTCCATGCTGGAAT-3′;

*GAPDH* forward: 5′-ACCACAGTCCATGCCATCAC-3′;

*GAPDH* reverse: 5′-TCCACCACCCTGTTGCTGTA-3′.

A GeneAmp PCR 9700 and a Veriti thermal cycler (Thermo Fisher Scientific) were used for PCR, and the amplified DNA fragments were separated using agarose gel electrophoresis. Then, they were stained with ethidium bromide and detected with a UV-transilluminator (ATTO, Tokyo, Japan). The expression levels of *BMCC1* in SK-N-AS cells were measured by quantitative real-time PCR (Q-PCR) using ABI PRISM 7500 system (Thermo Fisher Scientific). TaqMan probes for *BMCC1* (Hs00322421_m1) and *GAPDH* (4310884E) were purchased from Thermo Fisher Scientific.

### Immunoblotting

Whole cell lysate were immunoblotted as previously described [5]. The primary antibodies used for immunoblotting were described as follows. The rabbit antibody against human BMCC1 was generated by MBL (Nagoya, Japan) [5]. The Anti-E2F1 (#3742), anti-phospho-ATM (Ser1981) (#4526), anti-phospho-Chk2 (Thr68) (#2661), anti-phospho-H2A.X (Ser139) (#9718), anti-phospho-FOXO3a (Thr32) (#9464), anti-FOXO3a (#2497), anti-PARP (#9542, Fig. 5a and b) and anti-caspase-9 (#9502), anti-Bim (#4582), HRP-conjugated anti-Rabbit (#7074) and anti-Mouse (#7076) secondary antibodies were purchased from Cell Signaling Technology (Danvers, MA). Anti-Human phospho-E2F1 (pS364) was purchased from ROCKLAND (Limerick, PA) and anti-p73 (ab-3) was obtained from Millipore (Billerica, MA), whereas anti-ATM (sc-23,921), anti-PARP-1 (sc-8007), and anti-Actin (sc-8432) were bought from Santa Cruz Biotechnology (Dallas, TX).

### Luciferase reporter assay

Luciferase reporter plasmids harbored human *BMCC1* promoter region from − 1727 to + 78 (pGL3-*BMCC1-luc* promoter luciferase vector), a deletion mutant of *BMCC1* promoter (pGL3-*BMCC1-luc* △S1/S7 (△ − 1727/− 532)), and two point mutants of putative E2F1 binding constructs (pGL3-BMCC1-luc-△S1/S7 + S8 mut (− 329/− 322) and pGL3-BMCC1-luc-△S1/S7 + S9 mut (− 9/− 2)) (Fig. 3a). The luciferase reporter assay follows a previously described protocol [6].

### In vitro caspase cleavage assay

Proteins were synthesized from the T7 promoter on pcDNA6.2-BMCC1 or pcDNA3.1-MDM2 plasmids using in vitro transcription and translation (TNT) kit (Promega). To label this protein with biotin, TNT reaction was performed in the presence of biotinylated lysine-tRNA (Promega). Biotinylated proteins reacted with recombinant active caspase-9 (Millipore, Billerica, MA) or active caspase-3 (Millipore), supplied for SDS-PAGE, transferred onto a PVDF membrane, and detected using HRP-conjugated Streptavidin. Developed signals were captured using a LAS-4000 imager.

### Statistical analysis

All quantitative experiments were replicated at least three times independently. Error bars indicated standard deviation (SD). Statistical analysis was done by student t-test and *p*-value was calculated using Excel software (v14.7.3) (Microsoft, Redmond, WA).

## Results

### Expression profile of BMCC1 in apoptotic SK-N-AS cells induced by CDDP treatment

**Fig. 1:**
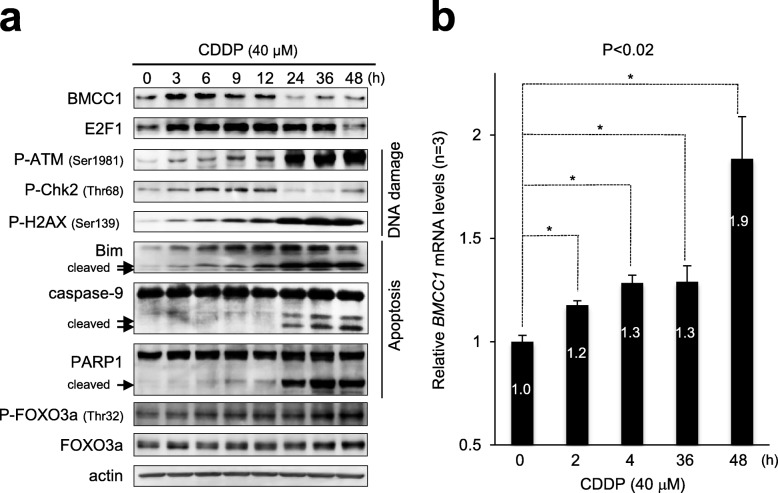
Expression profile of BMCC1 in apoptotic NB cells after CDDP treatment. **a** SK-N-AS cells harboring mutant p53 were treated with CDDP (at a final concentration of 40 μM) for indicated time intervals. After treatment, the cell lysates were prepared and immunoblotted. **b** The total RNA prepared from the indicated cells was analyzed using Q-PCR. Mean values were calculated from quadruplicate experiments. Error bars indicate means ± SD (*P < 0.02, *n* = 3)

We previously reported that BMCC1 facilitates apoptosis by neglecting multiple steps in Akt-survival pathway, such as anti-apoptotic Bcl-2 binding, inhibiting Akt and FOXO3a phosphorylation, and inducing Bim using the C-terminus BNIP-2 homology domain in human cells [5]. We also reported that acute accumulation of BMCC1 in human cells as a result of administration of DNA-damaging drugs, such as Cisplatin (CDDP) and Adriamycin (ADR), was needed for subsequent apoptosis progression [5]. However, expression profile of BMCC1 and its regulatory mechanisms during DNA damage-triggered apoptosis were unknown. Therefore, we first analyzed protein expression patterns of BMCC1 in SK-N-AS cells for up to 48 h after CDDP treatment (Fig. 1a). Consistent with our previous findings, rapid induction of BMCC1 was accompanied by initiation of DNA damage response, including phosphorylation of ATM at Ser-1981 (p-ATM), Chk2 at Thr-68 (p-Chk2), and H2A.X at Ser-139 (p-H2A.X) (Fig. 1a). Similar results were obtained in NBL-S cells (Additional file 1: Figure S1). In SK-N-AS cells with elevated expression of BMCC1, Bim induction was used to monitor CDDP-initiated progression toward apoptosis. BMCC1 protein expression decreased in SK-N-AS cells at a later step of apoptosis with elevated cleavage of caspase-9 and PARP-1 (24 to 48 h). It should be noted that a reduced expression of BMCC1 occurred along with accumulation of small fragments of pro-apoptotic Bim (15- and 12-kDa) that was mediated through cleavage by caspase-3 [26]. *BMCC1* expression at mRNA levels measured by Q-PCR was also significantly increased in cells shortly after CDDP treatment (2 and 4 h) (Fig. 1b). In contrast to reducing amount of BMCC1 at protein levels, sustained expression of *BMCC1* at mRNA levels was observed in apoptosis-progressed SK-N-AS cells at 36 and 48 h of CDDP treatment (Fig. 1b). Similarly, BMCC1 down-regulation was observed in NLF and LNCaP cells at 48 h of CDDP treatment in a dose-dependent manner (Additional file 2: Figures S2a and b). Furthermore, up-regulated expression of *BMCC1* at mRNA levels in apoptotic cells was also confirmed in LNCaP cells (Additional file 2: Figure S2b). It should be noted that reduction in the viabilities of SK-N-AS, NLF, and LNCaP cells after 48 h of CDDP treatment was confirmed by WST assay (Additional file 2: Figure S2c).

### Acute induction of BMCC1 was associated closely with accumulation of E2F1 in cells with DNA damage

**Fig. 2:**
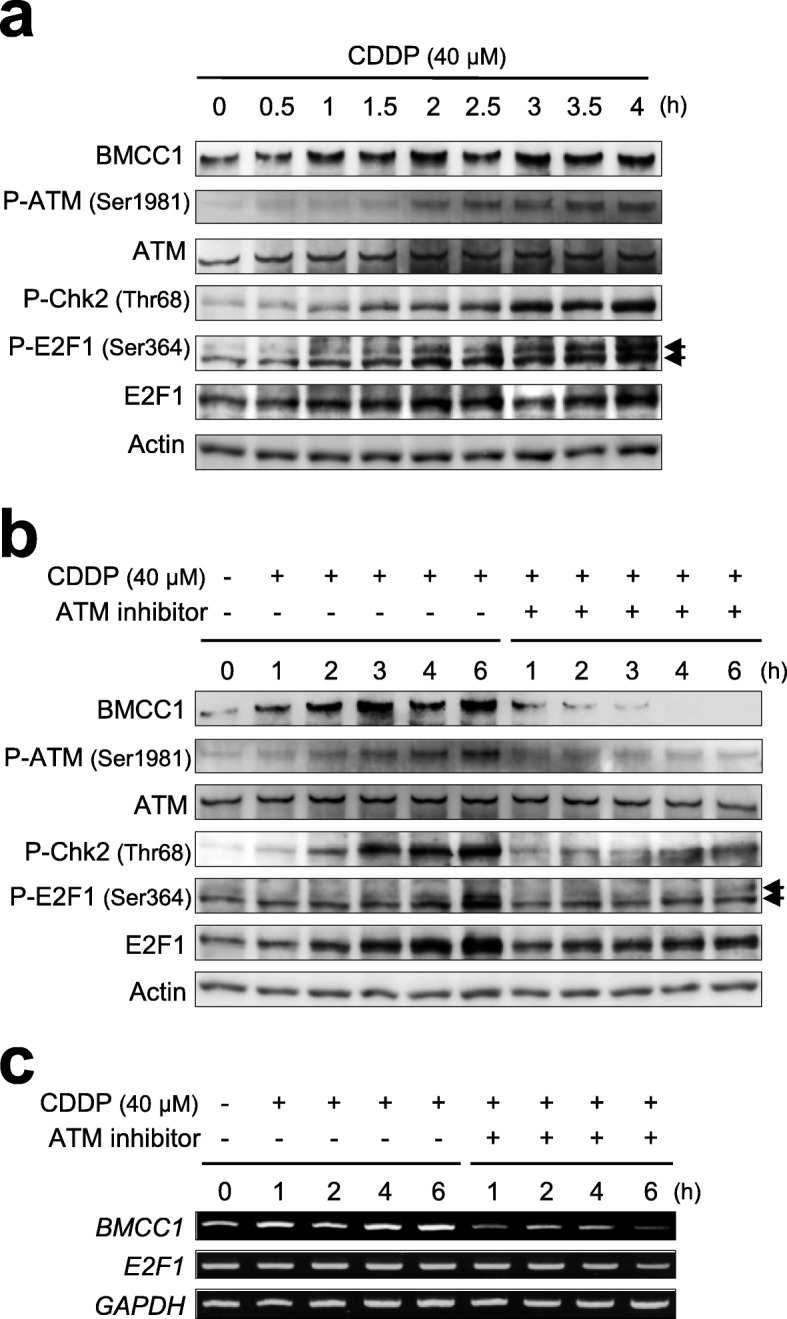
Induction of BMCC1 following CDDP treatment in NB cells was positively correlated with phosphorylation of ATM. **a** CDDP stimulates the expression of BMCC1 while promoting the phosphorylation of ATM, Chk2, and E2F1. SK-N-AS cells were treated with 40 μM of CDDP. At regular time intervals after treatment, the cell lysates were prepared and immunoblotted. **b** and **c** CDDP-dependent accumulation of BMCC1 was abrogated by ATM inhibition. CDDP-treated SK-N-AS cells were cultured in the presence or absence of an ATM inhibitor. At regular time intervals after incubation with the inhibitor, the cell lysates and total RNA were prepared and subjected to immunoblotting (**b**) and semiquantitative RT-PCR (**c**), respectively

During the early response to DNA damage, acute induction of BMCC1 was accompanied by initiation and accumulation of ATM signal pathway that includes p-ATM, p-Chk2, and p-H2A.X in SK-N-AS cells (Figs. 1a and 2a) [5]. On the other hand, induction of BMCC1 was cancelled when ATM activity was blocked using an ATM specific inhibitor, even in the presence of CDDP (Fig. 2b and c) [5]. Further research revealed that BMCC1 was trans-activated in an E2F1-dependent fashion in NB cells under normal culture conditions [6]. Therefore, these observations prompted us to examine whether the transactivation of *BMCC1* could be mediated by E2F1 even in apoptotic human cells initiated by CDDP-dependent DNA damage.

E2F1 promoted apoptosis, triggered by DNA-damaging agents, was mediated via accumulation through protein stabilization, not by transcriptional induction [8–12]. In addition to stabilization of E2F1, phosphorylation of E2F1 at Ser-364, catalyzed by Chk2 after phosphorylation by ATM/ATR, was associated with its transcriptional activity [13]. Indeed, up-regulation of E2F1 protein levels (and not mRNA levels) was detected in SK-N-AS cells shortly after CDDP treatment (Fig. 2a and b). Furthermore, phosphorylation of E2F1 at Ser-364 catalyzed by p-Chk2 was observed in these cells along with increased amounts of p-Chk2 (Fig. 2a and b). Again, when ATM-dependent DNA damage signal pathway was blocked in NB cells with an ATM inhibitor, there was a reduction in the immediate induction of BMCC1 both at mRNA and protein levels, normally followed by accumulation of E2F1 in response to DNA damage (Fig. 2b, c, Additional file 3: Figures S3a and b). On the other hand, ATM inhibitor influenced marginal effect on *E2F1* mRNA expressions in NB cells with DNA damage (Fig. 2c and Additional file 3: Figure S3b). These findings suggest that transcriptional induction of *BMCC1* mediated by DNA damage is closely associated with ATM-dependent stabilization of E2F1.

### ATM activity and an E2F-binding sight was required for activation of *BMCC1* promoter in response to DNA damage

**Fig. 3:**
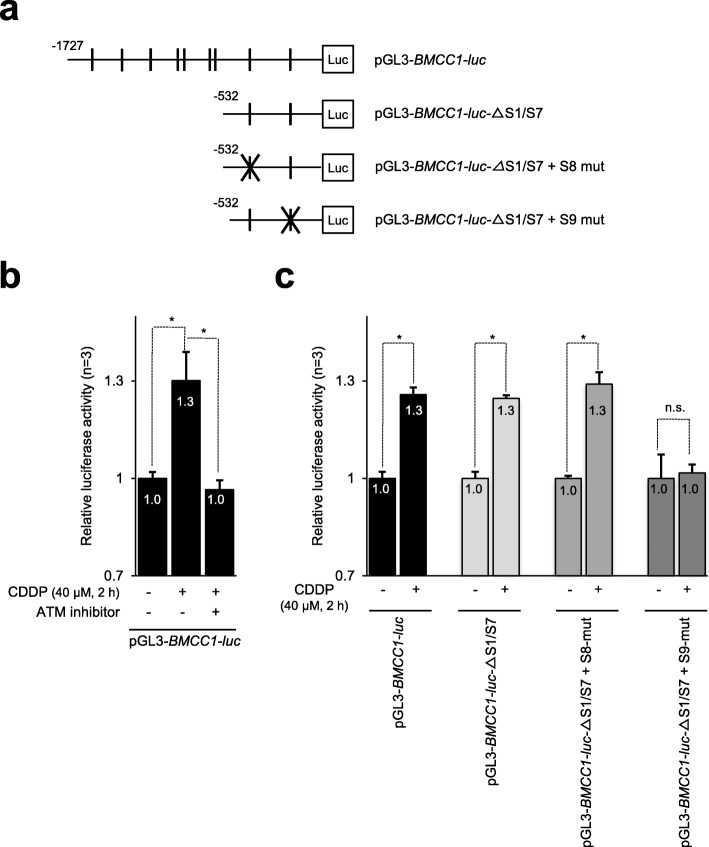
ATM activation and an E2F1 binding sequence were required for activation of *BMCC1* promoter in NB cells with DNA damage. **a** Schematic representation of *BMCC1* promoter constructs [6]. Vertical line indicated the position of E2F-binding sites. The E2F-binding site with X-mark harbors one base mutation to diminish the consensus sequence. **b** CDDP-mediated activation of *BMCC1* promoter is suppressed by ATM inhibitor treatment. pGL3-*BMCC1*-*luc* and pRL-TK (Renilla luciferase reporter plasmid) transfected SK-N-AS cells were maintained in normal medium for 46 h and then transferred in a fresh medium containing the appropriate ratios of CDDP and ATM inhibitor. Two hours after treatment, the luciferase activity was measured for all samples. Firefly luciferase activity was normalized to that of Renilla luciferase. Mean values were calculated from triplicate experiments. Error bars indicate standard deviation (*P < 0.01, n = 3). **c** The putative E2F-binding site S9 is responsible for the CDDP-dependent activation of *BMCC1* promoter. SK-N-AS cells were transfected with the luciferase reporter plasmids. Forty-six hours after transfection, the cells were cultured with or without CDDP. Two hours after treatment, luciferase activity was measured for all samples (*P < 0.001, *n* = 3). n.s. stands for not significant

Our earlier study demonstrated that an E2F-binding site-9 (S9) (Fig. 3a) is essential for E2F1-dependent activation of *BMCC1* promoter among nine putative E2F-binding sequences within a 1.7 kb upstream of the coding region [6]. Therefore, we wondered whether DNA damage could enhance the activity of *BMCC1* promoter through putative E2F-binding sequence(s) in NB cells. To this end, the promoter activity was measured using a luciferase assay [6]. Thus, SK-N-AS cells were transfected with luciferase reporter plasmid pGL3-*BMCC1*-l*uc* and Renilla luciferase reporter plasmid pRL-TK and cultured in the presence or absence of CDDP and/or ATM inhibitor.

CDDP treatment enhanced the luciferase activity driven by *BMCC1* promoter, but the enhancement was completely blocked in the presence of the ATM inhibitor (Fig. 3b). These results imply that CDDP-dependent activation of *BMCC1* promoter is strongly associated with ATM activity. In addition, by using deletion and mutant constructs of *BMCC1* promoter (ΔS1/S7, ΔS1/S7&S8-mut, and ΔS1/S7&S9-mut, Fig. 3a), it was demonstrated that the effect of CDDP treatment on *BMCC1* promoter depends on site-9 (Fig. 3c). This result was consistent with our previous finding that E2F binding site-9 was essential for *BMCC1* promoter activity [6]. Therefore, these results indicate that transcriptional induction of *BMCC1* mediated by DNA damage is regulated by E2F1-binding site S9 in its promoter region.

### E2F1 was essential for induction of BMCC1 in response to DNA damage

Next, we wondered whether acute induction of BMCC1 triggered by DNA damage is dependent on E2F1. To this end, we conducted shRNA-mediated knockdowns of *E2F1* in SK-N-AS cells, which were exposed to DNA damaging drugs.

**Fig. 4:**
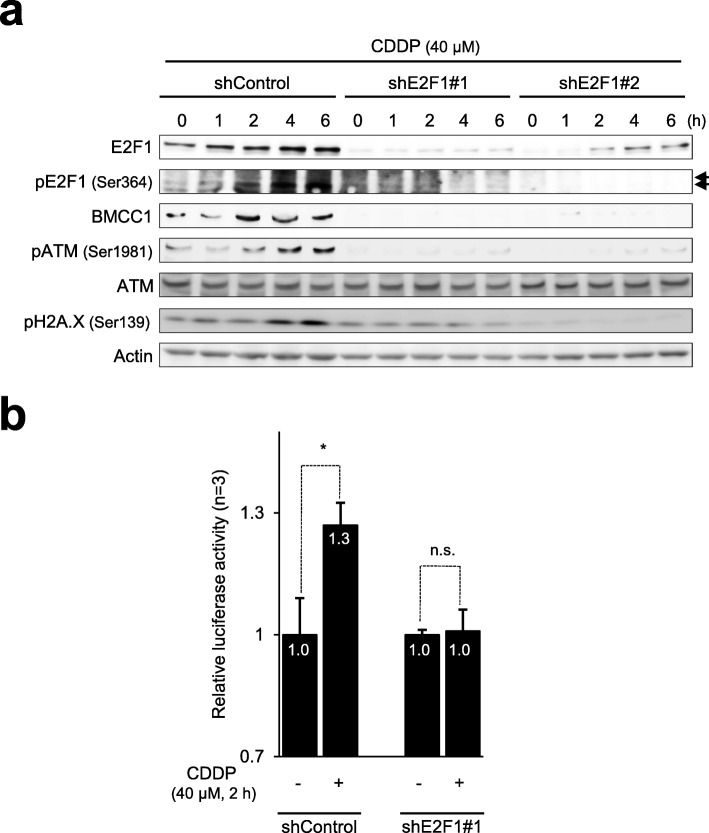
E2F1 is necessary for induction of BMCC1 with CDDP treatment. **a** Knockdown of E2F1 stops the accumulation of BMCC1 and phospho-ATM (p-ATM, ATM-S1981) in response to CDDP treatment. E2F1-depleted and non-depleted SK-N-AS cells were exposed to CDDP. Cell lysates were extracted at regular time intervals and analyzed by immunoblotting with the indicated antibodies. **b** Silencing of E2F1 diminishes CDDP-dependent activation of *BMCC1* promoter. E2F1-depleted and non-depleted SK-N-AS cells were transfected with pGL3-BMCC1-luc. Forty-eight hours after transfection, the cells were treated with CDDP or left untreated. Two hours after treatment, the activity of luciferase was measured for all samples. Data are shown as means ± SD (*P < 0.01, n = 3)

As shown in Fig. 4a, silencing of *E2F1* using shRNAs (shE2F1#1 and shE2F1#2) greatly limited the accumulation of E2F1, which was initiated by CDDP-mediated DNA damage. Consistent with E2F1 suppression, induction of BMCC1 facilitated by DNA damage was undetectable in *E2F1*-depleted cells. Similarly, the luciferase activity driven by *BMCC1* promoter increased in *E2F1*-non-depleted cells following CDDP exposure in comparison with that in untreated cells, whereas CDDP treatment had almost no effect on the luciferase activity in *E2F1*-depleted cells (Fig. 4b).

We further monitored DNA damage using elevated amounts of p-ATM, p-Chk2, and p-H2A.X. A significant attenuation of DNA damage caused by CDDP in *E2F1*-knockdown SK-N-AS cells was found (Fig. 4a). Because the accumulation of p-H2A.X is a marker for DNA double-strand breaks, the results suggest that knockdown of *E2F1* can attenuate the cellular response toward DNA double-strand breaks.

### Down-regulation of full-length BMCC1 in apoptotic cells through a caspase-9-dependent mechanism

**Fig. 5:**
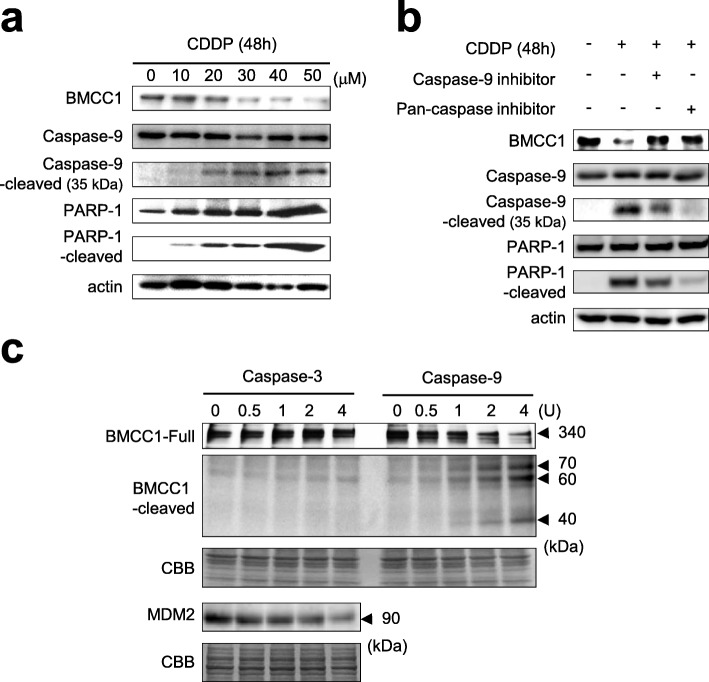
Cleavage of full-length BMCC1 during apoptosis was linked with activation of caspase-9. **a** Reduction of full-length BMCC1 was detected in apoptotic cells triggered by CDDP. SK-N-AS cells were treated with CDDP of various concentrations (0 to 50 μM). At 48 h after treatment, the harvested cells were immunoblotted. **b** Reduced amount of full-length BMCC1 by CDDP treatment was restored by inhibition of caspase-9. SK-N-AS cells were treated with 40 μM of CDDP for 24 h and cultured further in the presence or absence of 50 μM caspase inhibitors for 24 h. Harvested cells were analyzed by immunoblotting. **c** Cleavage of full-length BMCC1 by caspase-9 and not caspase-3 in vitro. Biotin-labeled full-length BMCC1 was synthesized using the TNT system and incubated with an active form of recombinant caspase-3 or caspase-9 at 37 °C for one hour. Protein degradation was detected using Streptavidin-HRP. Arrows indicate the full-length BMCC1 and three caspase-9-cleaved small bands. CBB staining of the gel was used as a loading control. MDM2 was cleaved by caspase-3 and used as a positive control of caspase-3 treatment

In addition to acute induction of BMCC1 in an E2F1-dependent manner during early stages of apoptotic process initiated by DNA damage, a subsequent down-regulation of BMCC1 protein in SK-N-AS cells undergoing apoptosis was also observed, due to accumulation of cleaved forms of caspase-9 and PARP-1 (Figs. 1a and 5a).

A previous in vitro study found that variants of BMCC1, known as BNIP family (e.g., BNIP-2 and BNIP-XL), could be cleaved efficiently by several caspases [24]. Therefore, it was assumed that full-length BMCC1 can also be cleaved by caspases during apoptosis. The potential amino acid sequences within full-length BMCC1 protein cleaved by caspase activity were searched, and two caspase-9 cleavage sites, LEID (660–663) and LEED (2380-2383), were identified (Additional file 4: Figure S4). The active caspase-9 can cleave proteins with LEXD sequence [27, 28], and thus the cleavage of full-length BMCC1 by caspase-9 in the apoptotic cells was verified. A pan-caspase inhibitor (z-VAD-FMK) and caspase-9-specific inhibitor (z-LEHD-FMK) were used to inhibit caspase activation during apoptosis initiated by CDDP. SK-N-AS cells were treated with 40 μM of CDDP and cultured in the presence or absence of caspase inhibitors for 24 h (Fig. 5b). Immunoblotting results revealed that pan-caspase and caspase-9-specific inhibitors efficiently block the accumulation of cleaved forms of caspase-9 and PARP-1 in SK-N-AS cells treated with CDDP. At the same time, we found that the reduced expression of BMCC1 in the cells treated with CDDP was significantly reversed by inhibition of caspase-9. These observations indicate that reduced expression of full-length BMCC1 within apoptotic cells was strongly associated with caspase-9 activation.

### Proteolytic cleavage of full-length BMCC1 by caspase-9

Finally, we investigate whether caspase-9 could directly cleave full-length BMCC1. To this end, biotin-labeled full-length BMCC1 was synthesized using TNT system [29] and subjected to in vitro caspase proteolysis assay (Fig. 5c). As expected, the full-length BMCC1 (340 kDa) was reduced efficiently in a dose-dependent manner following incubation with a recombinant active caspase-9. The smaller fragments of 70, 60, and 45 kDa bands were observed as a result of the treatment with active caspase-9. In addition, the recombinant active caspase-3 could not cleave full-length BMCC1, while it cleaves its target MDM2 [29]. Therefore, it was affirmed that the full-length BMCC1 is proteolytically cleaved by caspase-9 during apoptosis.

## Discussion

**Fig. 6:**
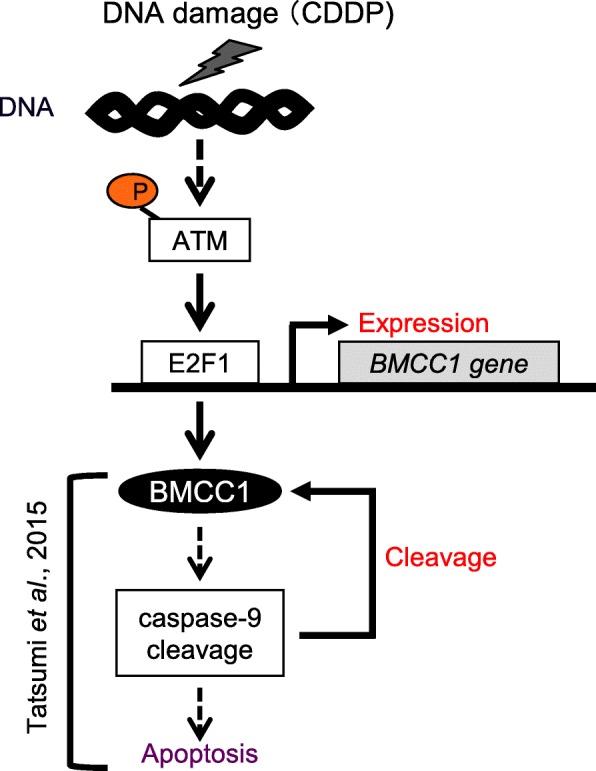
Proposed model for programmed expression of pro-apoptotic BMCC1 during apoptosis, triggered by DNA damage in NB cells. ATM- and E2F1-dependent transcriptional induction of *BMCC1* was identified in NB cells exhibiting early steps of apoptosis caused by DNA damage with short time treatment of CDDP. Subsequently, full-length BMCC1 was proteolytically cleaved by caspase-9, which was activated in the advanced steps of apoptosis, in NB cells. Our previous study demonstrated that BMCC1 promotes apoptosis through cleavage-dependent activation of caspase-9 in human cells

In this study, the programmed expression pattern of full-length BMCC1 during apoptosis triggered by a DNA-damaging drug CDDP in NB cells was identified (Fig. 6). Acute induction of BMCC1 in response to DNA damage was mediated by ATM-E2F1 by a transcription-dependent mechanism. Furthermore, subsequent reduction of BMCC1 during the later steps of apoptosis was controlled by proteolytic cleavage of BMCC1 by caspase-9. The outcomes of this work were used to propose the mechanism by which BMCC1 initiates and promotes apoptosis of human cells in response to DNA damage.

Recently, we reported that acute expression of BMCC1 during apoptosis is initiated by DNA-damaging drugs, and is required for progression of apoptosis in human cells [5]. We also reported that *BMCC1* is transcriptionally up-regulated via transcription factor E2F1-dependent mechanism involving E2F binding site S9 in the cell cycle of NB cells [6]. Here, it was noticed that transcriptional induction of *BMCC1* during apoptosis is regulated by E2F1-dependent mechanism via E2F binding site S9, in association with accumulation of E2F1 after CDDP treatment. Furthermore, requirement of ATM activation for the E2F1-dependent transcriptional up-regulation of *BMCC1* in response to DNA damage in SK-N-AS cells was proven by inhibition of DNA damage response initiated by CDDP using an ATM inhibitor. As previously reported, overexpression of BMCC1 in human cells induces apoptosis by inhibiting multiple steps in Akt-survival signal and by DNA-damaging drugs [5]. Therefore, similar to pro-apoptotic function of p73 in cells with DNA damage [15–17], BMCC1 also functions in apoptosis initiated by DNA damage through elevated expression by E2F1. Hence, these findings point out that BMCC1 expression induced by an E2F1-dependent mechanism may act in sensitizing cells to DNA damage and eliminating cells with DNA damage(s) by promoting apoptosis.

In addition to E2F1 role in apoptosis of DNA-damaging cells, which is modulated downstream of ATM/ATR activation [8–13], E2F1 is involved in promoting DNA repair through phosphorylation of p53 and Chk2 by an ATM- and Nbs1-dependent mechanism [30, 31]. This is accompanied by the use of proteins involved in DNA repair, such as Mre11, Rad51, and Nbs1 (MRN) complex, at sites of DNA double-strand breaks [32, 33]. By contrary, we observed that knockdown of *E2F1* in SK-N-AS cells led to attenuated levels of p-ATM and p-H2A.X, two DNA double-strand break indicators in the early stages of apoptosis. Similarly, knockdown of *E2F1* in SK-N-AS cells inhibited the expression of BMCC1 in normal culture conditions [6], preventing even under DNA-damaging conditions.

In the later stages of apoptosis, reduced amounts of full-length BMCC1 were accompanied by cleavage of caspase-9 and PARP-1. Furthermore, in vitro experiments demonstrated that full-length BMCC1 was proteolytically cleaved by a recombinant active form of caspase-9. Therefore, these observations suggest that reduced expression of full-length BMCC1 in apoptotic cells was directly regulated by activated caspase-9. Although full-length BMCC1 can promote apoptosis through BCL2 binding and Akt inhibition with its BNIP-2 homology C-terminus [5], the fragment(s) of BMCC1 cleaved by caspase-9 may also acquire new functions during apoptosis progression. Thus, in well agreement with previously reported results on BH3-containg pro-apoptotic proteins, such as Bid [34], Bad [35] and Bim [26], which enhance apoptotic cascade, a pro-apoptotic role of caspase-cleaved fragments with BH3 domain of BNIP family proteins, including BMCC1, was identified in this study.

From a clinical point of view, BMCC1 is considered as a tumor suppressor. BMCC1 is expressed widely in normal cells, but its expression is decreased in skin, prostate, and colon cancers [5]. Attenuated expression of *BMCC1* is associated with aggressive NB, and thus it is a prognostic indicator of NB [3]. In addition, BMCC1 has a clear effect in suppressing the prostate cancer [36]. Furthermore, loss of functional mutations in the coding region of *BMCC1* has been identified in merkel cell carcinoma [37] and parathyroid carcinoma [38], but the functional role of these mutations in promoting apoptosis initiated by DNA damage remains to be elucidated.

## Conclusions

The present study uncovered the programmed expression (E2F1-dependent transcriptional induction and caspase-9-dependent proteomic cleavage) of full-length BMCC1 in the course of apoptosis triggered by DNA damage. Our current findings may provide greater insight into tumor suppressive functions, such as sensitivity to DNA-damaging agents and spontaneous regression of BMCC1 in NB and other cancers.

## Additional files

Additional file 1: **Figure S1.** Induction of BMCC1 following CDDP treatment in NBL-S cells. Immunoblot results demonstrated that induced expression of BMCC1 in NBL-S cells was detected after the treatment with 20 μM of CDDP with indicated time points. Increase in the phosphorylation of ATM, Chk2, and E2F1 was concurrently observed. p73, which is induced in response to DNA damage and is controlled by E2F1, was employed as a positive control of the experiment. (PPTX 334 kb)

Additional file 2: **Figure S2**. Reduced expression of full-length BMCC1 in apoptotic NB and non-NB cells induced by CDDP. NB-derived NLF cells (a) and Prostate cancer-derived LNCaP cells (b) were treated with CDDP at various concentrations. At 48 h after treatment, harvested cells were immunoblotted (a and b, upper panels). *BMCC1* mRNA expression in CDDP-treated LNCaP cells was analyzed by semi-quantitative RT-PCR. The *GAPDH* mRNA level was used as the loading control (b, lower panels). (c) Viabilities of SK-N-AS, NLF and LNCaP cells were measured by WST-8 assay after treatment with CDDP for 48 h at the indicated concentrations. Data represent the mean ± SD of six independent experiments. (PPTX 214 kb)

Additional file 3: **Figure S3.** CDDP-mediated induction of BMCC1 in NBL-S cells carrying wild-type *p73*. CDDP-dependent transcriptional activation of *BMCC1* is blocked by the treatment with ATM inhibitor. NBL-S cells were treated with 20 μM of CDDP in the presence or absence of ATM inhibitor. At the indicated time periods after the treatment, whole cell lysates were immunoblotted (a) and total RNA was prepared and analyzed by semi-quantitative RT-PCR (b). Transcriptional activation of *p73* in response to DNA damage was mediated by ATM-E2F1 and was used for a positive control of the experiment (b). (PPTX 752 kb)

Additional file 4: **Figure S4.** Predicted caspase-9 cleavage sites. Schematic model of BMCC1 protein. Arrows indicate the predicted cleavage sites of caspase-9. (PPTX 47 kb)

## Data Availability

All data obtained from this study are included in this article.

## Abbreviations

ATM: Ataxia telangiectasia mutated
ATR: ATM and RAD3-related
BCH: BNIP2 and Cdc42GAP homology
BH3: BCL2-homology domain 3
BMCC1: BNIP2 and Cdc42GAP homology motif-containing molecule at the carboxyl terminal region 1
CDDP: Cisplatin
FBS: Fetal bovine serum
FOXO3a: Forkhead box O3a
MRN: Mre11, Rad51 and Nbs1
NB: Neuroblastoma
NGF: Nerve growth factor
PARP1: Poly [ADP-ribose] polymerase 1
PI3K: Phosphoinositide 3-kinase
RT-PCR: Reverse transcription polymerase chain reaction
