# Supplementary figures and images for: Programmed expression of pro-apoptotic BMCC1 during apoptosis, triggered by DNA damage in neuroblastoma cells
Source: BMC Cancer. 2019 Jun 6;19:542. doi: 10.1186/s12885-019-5772-4 (PMC6555734; doi:10.1186/s12885-019-5772-4)

## Slide 1
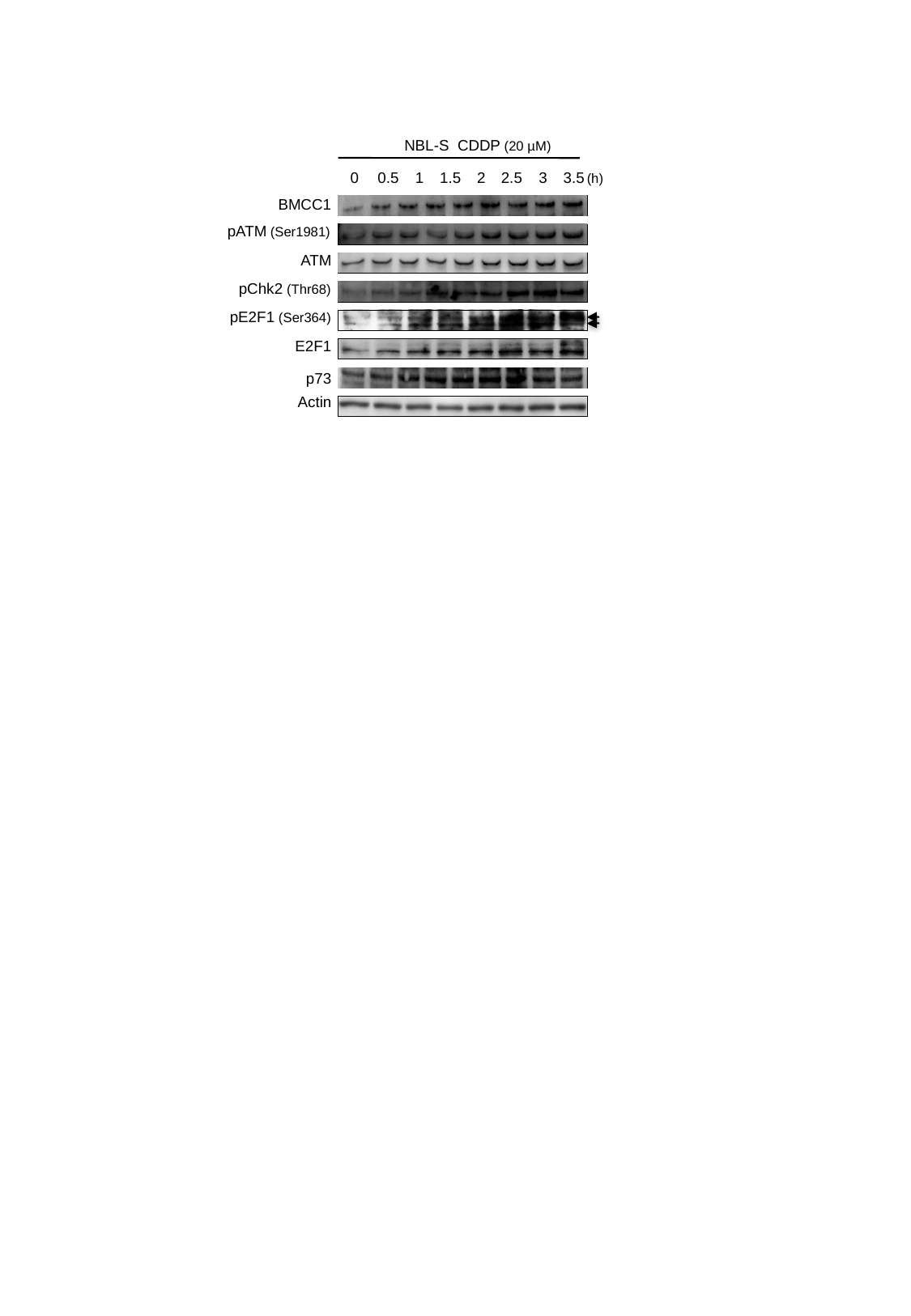

NBL-S CDDP (20 µM)
0
0.5
1
1.5
2
2.5
3
3.5
(h)
BMCC1
pATM (Ser1981)
ATM
pChk2 (Thr68)
pE2F1 (Ser364)
E2F1
p73
Actin

Supplement: Supplementary file 1 — Figure S1. Induction of BMCC1 following CDDP treatment in NBL-S cells. Immunoblot results demonstrated that induced expression of BMCC1 in NBL-S cells was detected after the treatment with 20 μM of CDDP with indicated time points. Increase in the phosphorylation of ATM, Chk2, and E2F1 was concurrently observed. p73, which is induced in response to DNA damage and is controlled by E2F1, was employed as a positive control of the experiment. (PPTX 334 kb) [file 12885_2019_5772_MOESM1_ESM.pptx]

## Slide 1
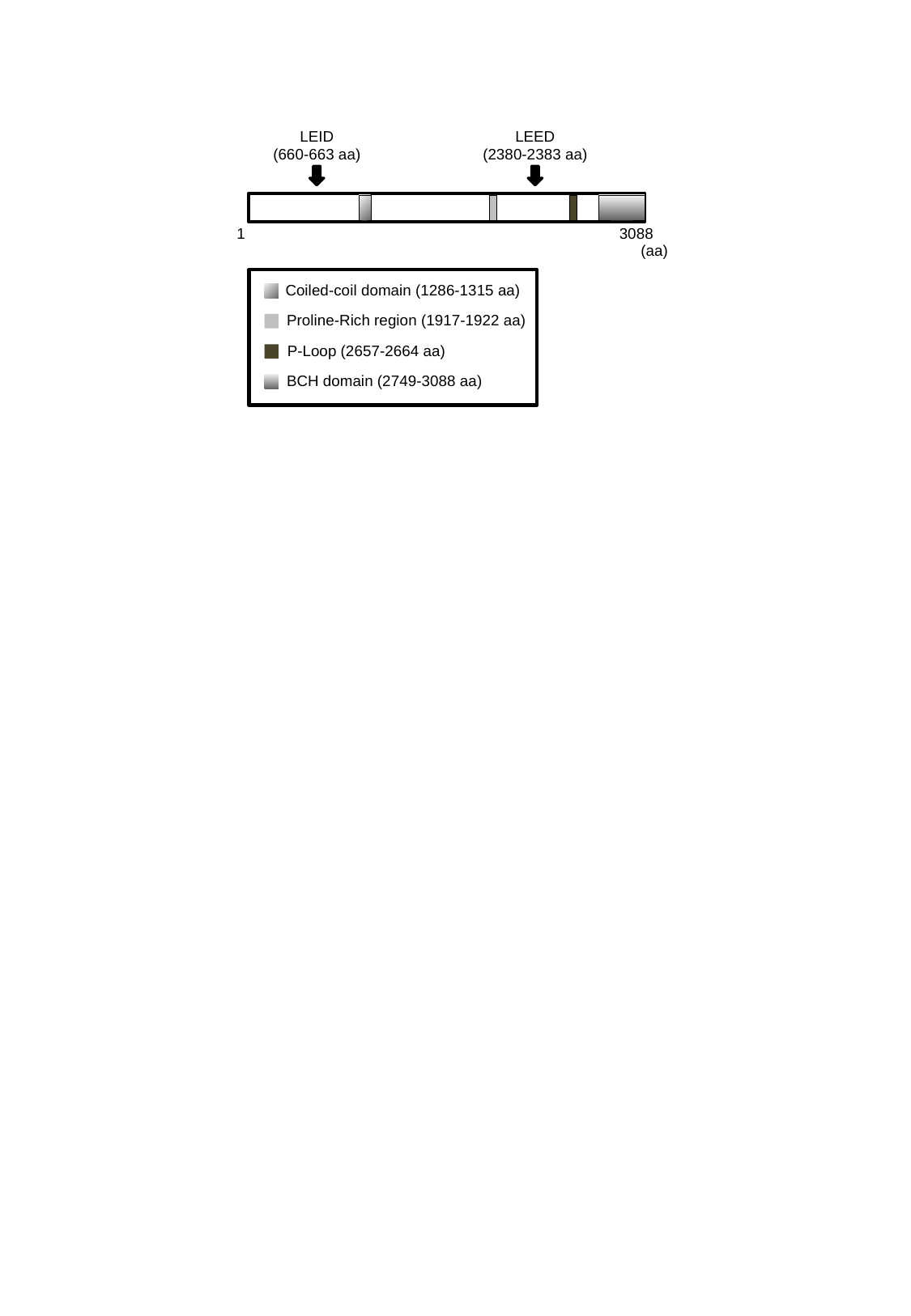

LEID
(660-663 aa)
LEED
(2380-2383 aa)
1
3088
(aa)
Coiled-coil domain (1286-1315 aa)
Proline-Rich region (1917-1922 aa)
P-Loop (2657-2664 aa)
BCH domain (2749-3088 aa)

Supplement: Supplementary file 4 — Figure S4. Predicted caspase-9 cleavage sites. Schematic model of BMCC1 protein. Arrows indicate the predicted cleavage sites of caspase-9. (PPTX 47 kb) [file 12885_2019_5772_MOESM4_ESM.pptx]
