# Supplementary material for: Programmed expression of pro-apoptotic BMCC1 during apoptosis, triggered by DNA damage in neuroblastoma cells
Source: BMC Cancer. 2019 Jun 6;19:542. doi: 10.1186/s12885-019-5772-4 (PMC6555734; doi:10.1186/s12885-019-5772-4)
Supplement: Supplementary file 2 — Figure S2. Reduced expression of full-length BMCC1 in apoptotic NB and non-NB cells induced by CDDP. NB-derived NLF cells (a) and Prostate cancer-derived LNCaP cells (b) were treated with CDDP at various concentrations. At 48 h after treatment, harvested cells were immunoblotted (a and b, upper panels). BMCC1 mRNA expression in CDDP-treated LNCaP cells was analyzed by semi-quantitative RT-PCR. The GAPDH mRNA level was used as the loading control (b, lower panels). (c) Viabilities of SK-N-AS, NLF and LNCaP cells were measured by WST-8 assay after treatment with CDDP for 48 h at the indicated concentrations. Data represent the mean ± SD of six independent experiments. (PPTX 214 kb) [file 12885_2019_5772_MOESM2_ESM.pptx]

## Slide 1
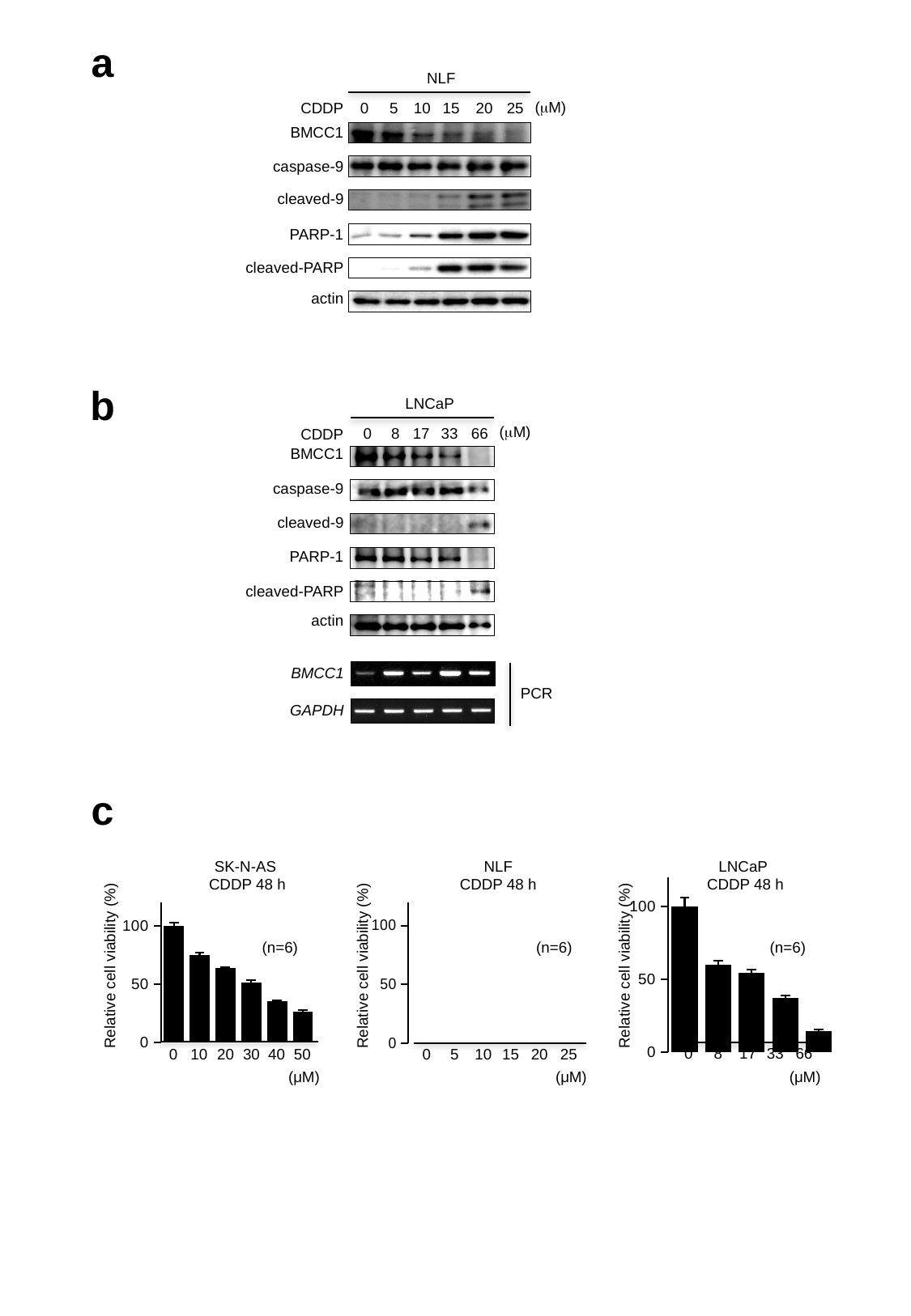

a
NLF
(mM)
CDDP
0
5
10
15
20
25
BMCC1
caspase-9
cleaved-9
PARP-1
cleaved-PARP
actin
b
LNCaP
(mM)
0
8
17
33
66
CDDP
BMCC1
caspase-9
cleaved-9
PARP-1
cleaved-PARP
actin
BMCC1
PCR
GAPDH
c
SK-N-AS
CDDP 48 h
 NLF
CDDP 48 h
LNCaP
CDDP 48 h
### Chart
| Category | |
|---|---|
### Chart
| Category | |
|---|---|
| 0.0 | 100.0 |
| 2.5 | 60.35083819205155 |
| 5.0 | 54.65375948586804 |
| 10.0 | 37.36244859189794 |
| 20.0 | 14.75601992663925 |
### Chart
| Category | |
|---|---|
| 0μM | 99.99999733009267 |
| 5μM | 99.75864932516578 |
| 10μM | 86.55252402359865 |
| 15μM | 75.12601806547089 |
| 20μM | 38.4854025044494 |
| 25μM | 27.39264188440053 |Relative cell viability (%)
Relative cell viability (%)
Relative cell viability (%)
(n=6)
(n=6)
(n=6)
0
8
17
33
66
0
10
20
30
40
50
0
5
10
15
20
25
(μM)
(μM)
(μM)
