# Supplementary material for: Programmed expression of pro-apoptotic BMCC1 during apoptosis, triggered by DNA damage in neuroblastoma cells
Source: BMC Cancer. 2019 Jun 6;19:542. doi: 10.1186/s12885-019-5772-4 (PMC6555734; doi:10.1186/s12885-019-5772-4)
Supplement: Supplementary file 3 — Figure S3. CDDP-mediated induction of BMCC1 in NBL-S cells carrying wild-type p73. CDDP-dependent transcriptional activation of BMCC1 is blocked by the treatment with ATM inhibitor. NBL-S cells were treated with 20 μM of CDDP in the presence or absence of ATM inhibitor. At the indicated time periods after the treatment, whole cell lysates were immunoblotted (a) and total RNA was prepared and analyzed by semi-quantitative RT-PCR (b). Transcriptional activation of p73 in response to DNA damage was mediated by ATM-E2F1 and was used for a positive control of the experiment (b). (PPTX 752 kb) [file 12885_2019_5772_MOESM3_ESM.pptx]

## Slide 1
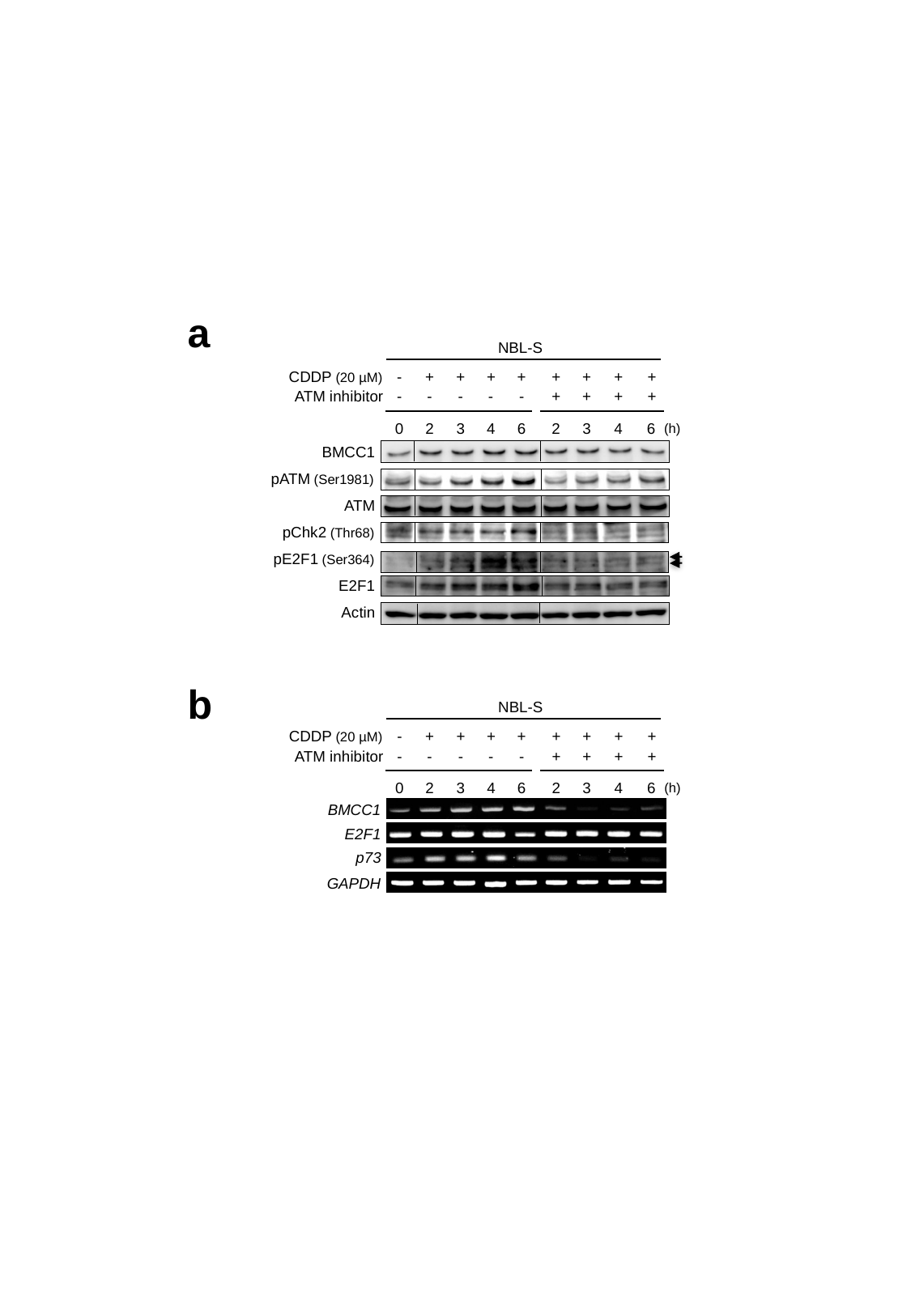

a
NBL-S
CDDP (20 µM)
-
+
+
+
+
+
+
+
+
ATM inhibitor
-
-
-
-
-
+
+
+
+
0
2
3
4
6
2
3
4
6
(h)
BMCC1
pATM (Ser1981)
ATM
pChk2 (Thr68)
pE2F1 (Ser364)
E2F1
Actin
b
NBL-S
CDDP (20 µM)
-
+
+
+
+
+
+
+
+
ATM inhibitor
-
-
-
-
-
+
+
+
+
0
2
3
4
6
2
3
4
6
(h)
BMCC1
E2F1
p73
GAPDH
